# A Patient with an Intradural Tumor: An Unexpected Finding

**DOI:** 10.7759/cureus.7376

**Published:** 2020-03-23

**Authors:** Devang Padalia, Allan R Escher, Nasrin N Aldawoodi, Neal Shah

**Affiliations:** 1 Anesthesiology/Pain Medicine, H. Lee Moffitt Cancer Center and Research Institute, Tampa, USA; 2 Anesthesiology, H. Lee Moffitt Cancer Center and Research Institute, Tampa, USA; 3 Anesthesia and Interventional Pain Management, H. Lee Moffitt Cancer Center and Research Institute, Tampa, USA

**Keywords:** retroperitoneal fibrosis, intrathecal drug delivery system, aspetic inflammatory mass, intrathecal granuloma, catheter-tip granuloma, intrathecal pump therapy, morphine, intrathecal complication, chronic pain, intradural tumor

## Abstract

Chronic back pain patients may require escalating doses of systemic opioids. In refractory cases, implantation of an intrathecal drug delivery system (IDDS) may provide effective relief of pain and improve overall function. This system infuses opioid directly into the cerebrospinal fluid via a catheter. While efficacious, it can be associated with complications, one of the most severe being the formation of a catheter-tip granuloma that can lead to permanent neurological deficits. We present a case of a 38-year-old male with an IDDS for pain related to retroperitoneal fibrosis, who began developing worsening back pain along with new-onset lower extremity weakness. A catheter-tip granuloma was suspected, and the patient was advised to obtain emergent spine imaging. He was non-compliant until the point of becoming wheelchair bound, whereupon imaging was finally obtained. Magnetic resonance imaging revealed an intradural mass causing spinal cord compression. After emergent surgical resection, pathology revealed a malignant tumor. Any patient with IDDS and escalating pain levels or new neurological deficits needs urgent neuroimaging to rule out catheter-tip granuloma. However, as this case demonstrates, the differential diagnosis should remain broad and always include neoplasm or abscess.

## Introduction

Intrathecal drug delivery system (IDDS) is efficacious in managing malignant and non-malignant chronic pain refractory to standard treatment [1]. Morphine via an IDDS provides analgesia by infusion of this opioid directly into the cerebrospinal fluid through an intrathecal catheter [2-4]. This analgesic technique reduces the incidence of adverse effects caused by systemic morphine administration, including nausea, vomiting, somnolence, and constipation [4]. There are numerous complications associated with IDDS, but formation of an intrathecal granuloma is one of the most catastrophic. A catheter-tip granuloma is an aseptic inflammatory mass associated with intrathecal opioid infusions and is believed to originate from the arachnoid layer [5]. The granuloma may block the infusion or can compress the spinal cord or exiting nerve roots, causing pain and corresponding neurological deficits [6]. It is usually diagnosed with a magnetic resonance imaging (MRI) or computerized tomography (CT) myelogram [7]. Once this mass forms, the treatment is usually halting the infusion or changing it to normal saline. In selected cases, it may necessitate catheter removal and surgical excision of the mass [8].

## Case presentation

A 38-year-old male with a history of chronic pain secondary to idiopathic retroperitoneal fibrosis (RPF) was referred to our tertiary cancer pain clinic for pain management. The pain started in his low back and primarily radiated across his abdomen and into his lower extremities. His abdominal and pelvic MRI showed an enhancing retroperitoneal mass surrounding the aorta with compression of the ureters and inferior vena cava.

After a complete evaluation, the patient was placed on oral opioids, including methadone and hydromorphone, as well as adjuvant medication therapy, including baclofen and gabapentin. Despite this treatment, he continued to have intractable pain and a decision was made to offer an IDDS trial after a few months. The trial was successful, and he subsequently had a permanent IDDS implantation with morphine infusion. After about two years, his pain symptoms progressed and he was offered a spinal cord stimulator (SCS) as an off-label use for the intractable abdominal, pelvic, and back pain. SCS was implanted after the completion of a successful trial.

Over the course of treatment, his intrathecal medications were escalated to morphine 33.08 mg/day, clonidine 661 mcg/day, and baclofen 248 mcg/day. Initially, his pain was managed well, but he eventually showed declining functional status with progressive weakness in his lower extremities and increasing pain. At this point, it remained unclear whether his pain and neurological symptoms were due to catheter-tip granuloma, worsening RPF, or a non-functioning SCS. Therefore, a CT myelogram was ordered. The risks of paralysis were explained to the patient; however, he deferred imaging at that time. Later, the patient also reported that his SCS was causing pain at the implantation site and he wanted it removed. Since he began developing progressive neurological deficits, a decision was made to explant his non-MRI compatible SCS. After this surgery, the patient refused to obtain imaging studies as ordered. For unknown reasons, the patient deferred his MRI appointments as ordered.

**Figure 1:**
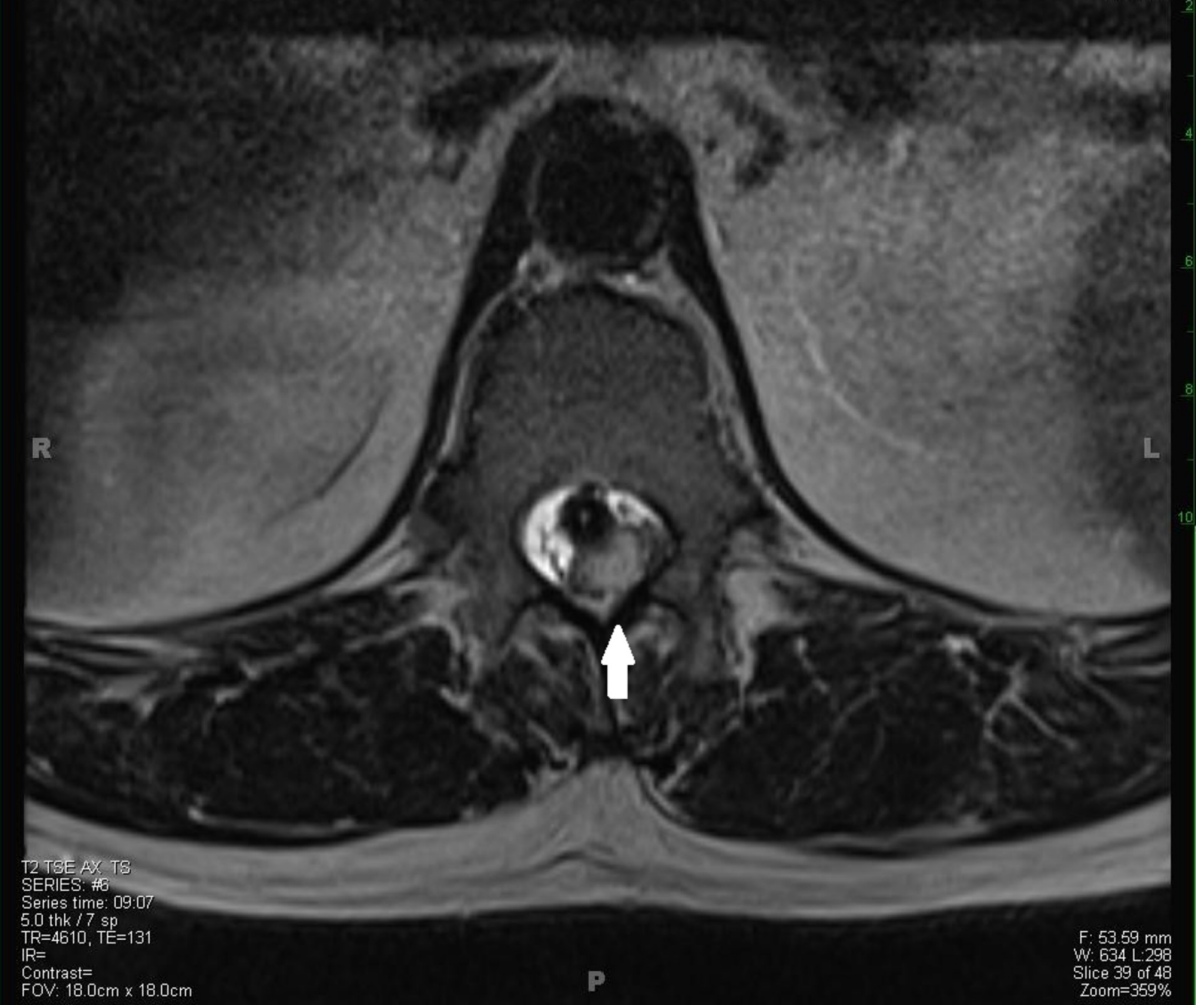
T2-weighted axial MRI showing a 2x1x1 cm intradural lesion at T11-T12 with impingement and compression of thoracic cord and associated edema producing a mass effect. T, thoracic.

**Figure 2:**
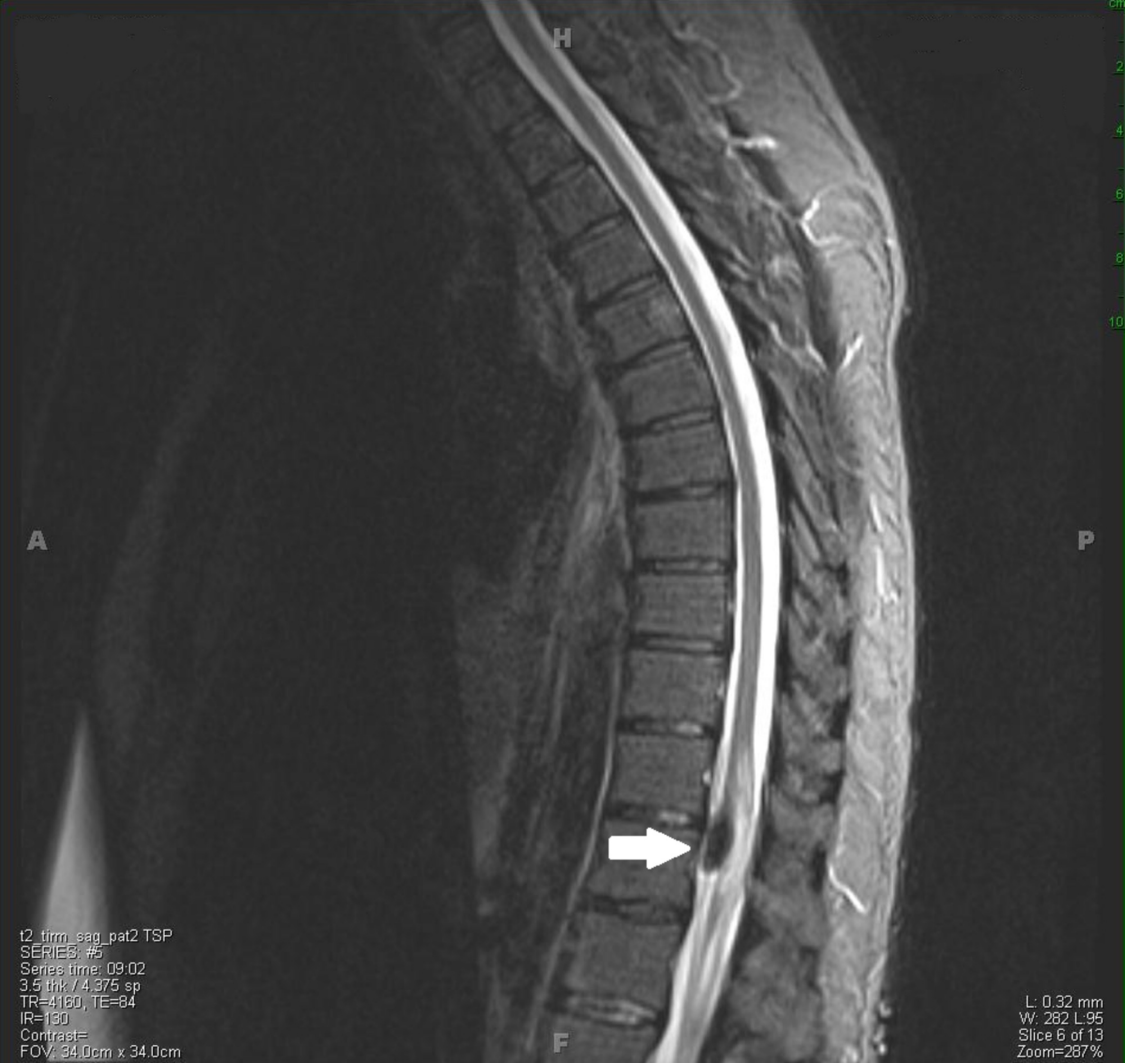
T2-weighted sagittal MRI displaying the intradural lesion.

**Figure 3:**
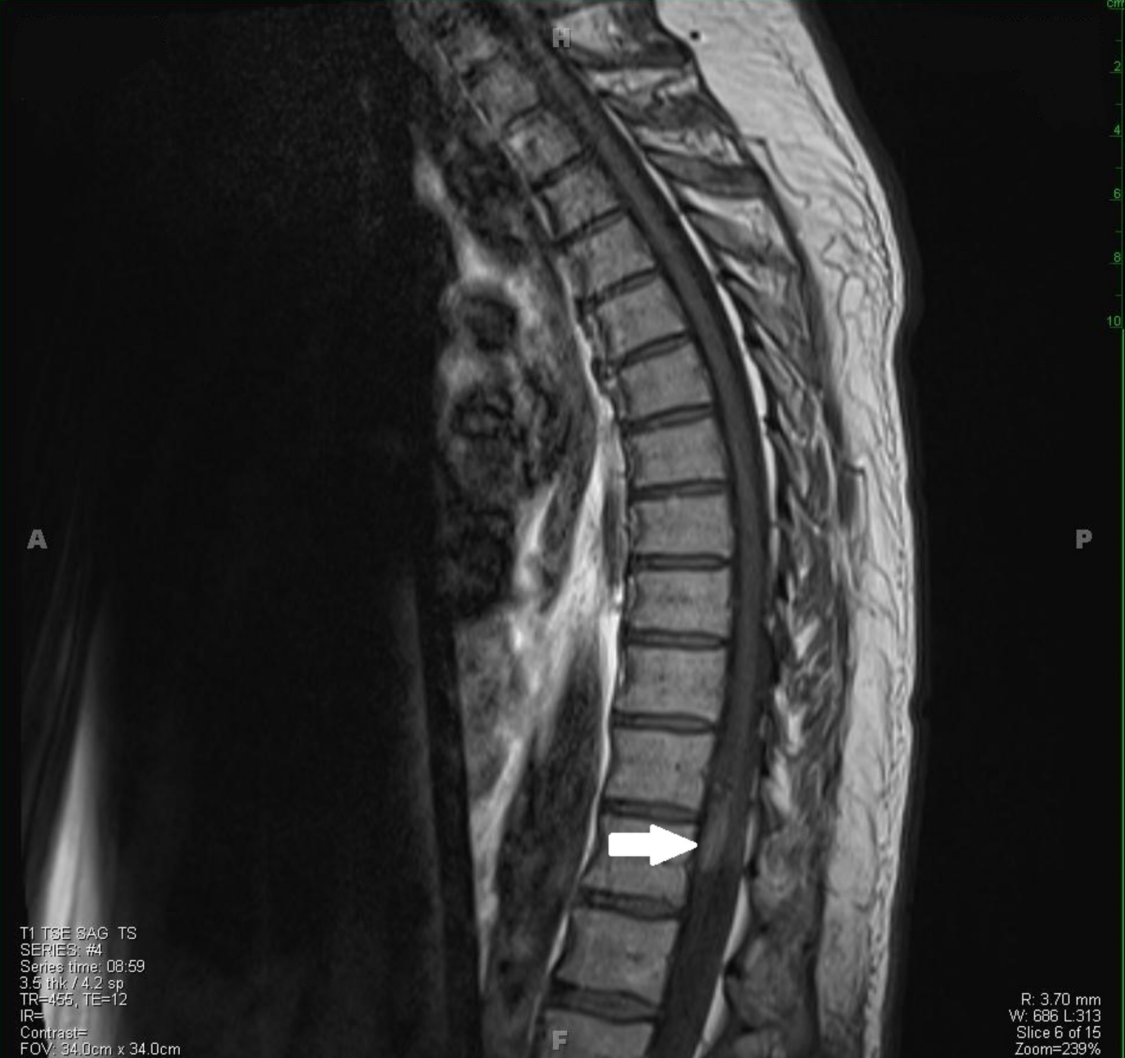
: T1-weighted sagittal MRI displaying the intradural lesion.

After 10 months of non-compliance, the patient presented with profound weakness of the lower extremities and was confined to wheelchair. Emergent MRI revealed an intradural lesion at T11-T12 with impingement upon, and compression of, the thoracic cord, spinal cord edema, and associated mass effect (Figures 1-3).

Neurosurgery performed an emergent thoracic laminectomy at T11-12 with intradural exploration. The pathology report confirmed an intradural malignant epithelial round/spindle cell neoplasm with abundant myxoid matrix. The patient was discharged to a neurosurgical rehabilitation facility. He later received radiation therapy for possible residual tumor. Fortunately for this patient, there was a gradual improvement in his neurological status as well as pain control.

## Discussion

Chronic pain causes significant economic impact on society through health care utilization, lost productivity, and disability [4]. Management of chronic pain should include multimodal approaches: physical therapy, cognitive and behavioral therapies (e.g., biofeedback), pharmacotherapy, and correction of mood disorder [3,4]. After conventional non-invasive pain regimen has failed, IDDS may be useful in managing intractable malignant and non-malignant chronic pain [3].

RPF is a rare cause of chronic low back pain with a prevalence of about 1.3 per 100,000 population and includes a range of diseases characterized by proliferation of aberrant fibro-inflammatory tissue [9]. Proliferation of fibrous tissue in the retroperitoneum can lead to obstruction in the ureters, kidneys, aorta, and other structures. Patients usually present with low back pain, and diagnosis is difficult because of the non-specific nature of the symptoms. If diagnosed and treated early, some cases have good prognosis but other aggressive forms have a mean survival of less than six months [9,10]. Management of the pain from this condition is typically with oral opioids. Although RPF is not malignant in nature, it can be considered "malignant" due to decreased life expectancy/survival; hence, an IDDS may be a reasonable option for these patients.

One of the complications of opiates in IDDS is the formation of a catheter-tip granuloma, which is an aseptic inflammatory mass originating from the arachnoid layer [5]. It has been proposed that the opiate acts as a mitogen that activates a protein kinase cascade causing inflammation through lymphocyte activation [10]. Other proposed mechanisms include the effect of opioids on endothelial cells, granulocytes, and monocytes that release nitric oxide causing monocyte migration [11]. Opioids may also cause cytokine formation leading to an inflammatory response [12]. This reduces cerebrospinal fluid-flow velocity further amplifying the drug concentration in the area and leading to granuloma formation. This cord-compressing lesion should always be suspected when patients present with progressive weakness, pain, or numbness in the distribution of the compressed nerves or spinal cord, loss of pain control from the IDDS, or increasing requirements of the pain medication [13]. A contrast-enhanced T1-weighted MRI or CT myelogram would show an enhancing mass lesion around the tip of the catheter [7]. Failure to diagnose this condition at an early stage can lead to permanent neurological injury. It is suspected that the use of high-dose opioids, specifically morphine, leads to granuloma formation. That is why a broad consensus exists that intrathecal opioids in IDDS should be prescribed at lowest effective dose and at the lowest concentration possible to prevent this complication [14]. The IDDS should be stopped and serial MRIs are done to observe the regression of the granuloma. Once it is resolved, a low-dose therapy can be restarted and the patient is monitored closely for reoccurrence. If the neurological symptoms persist, options include spinal cord decompression, excision of the granuloma, and catheter removal [7].

Catheter-tip granuloma remains one of the common causes of a mass lesion in IDDS. Imaging studies such as MRI or CT myelogram should provide clarity with lesion diagnosis. Important differential diagnoses should include intramedullary lesions such as neoplasms, extramedullary lesions (e.g. meningioma), as well as epidural lesions (e.g. abscess).

Intradural spinal tumors are very rare with an annual incidence of two to four per 1,000,000 population [15]. Tumors may present with varied neurological deficits depending on their location and can cause radicular pain, cord compression, and loss of bowel or bladder function. Treatment options for intradural tumors include open microsurgery to establish a histopathological diagnosis and to decompress the neural structures [15-17]. In patients with benign tumors, such as meningiomas and schwannomas, complete tumor resection should be aimed for to reduce the risk of tumor recurrence [17,18].

## Conclusions

Chronic pain patients are both challenging and rewarding to treat. The signs and symptoms of neurological compromise may be overlooked by the busy practitioner. Any new signs and symptoms should be approached with caution and investigated to discover a cause. Vigilance in patients with an opioid IDDS should prompt careful monitoring to detect any complications. In this case, a catheter-tip granuloma from an IDDS was suspected and investigated. Instead, the discovery of a rare intradural tumor (malignant epithelial round/spindle cell neoplasm) proved an unexpected finding. After surgery, rehabilitation, and radiation therapy, our patient experienced improvement in both function and pain control.

## References

[1] Jain S, Malinowski M, Chopra P, Varshney V, Deer TR (2019). Intrathecal drug delivery for pain management: recent advances and future developments. Expert Opin Drug Deliv.

[2] Deer TR, Malinowski M, Varshney V, Pope J (2019). Choice of intrathecal drug in the treatment of neuropathic pain: new research and opinion. Expert Rev Clin Pharmacol.

[3] Ruan X (2007). Drug-related side effects of long-term intrathecal morphine therapy. Pain Physician.

[4] Knight KH, Brand FM, Mchaourab AS, Veneziano G (2007). Implantable intrathecal pumps for chronic pain: highlights and updates. Croat Med J.

[5] Yaksh TL, Allen JW, Veesart SL (2013). Role of meningeal mast cells in intrathecal morphine evoked granuloma formation. Anesthesiology.

[6] McMillan MR, Doud T, Nugent W (2003). Catheter-associated masses in patients receiving intrathecal analgesic therapy. Anesth Analg.

[7] Magill ST, Wang P, Eller JL, Burchiel KJ (2008). Differentiating intrathecal catheter tip granulomas from normal magnetic resonance image distortion caused by metallic catheter tips. Neurosurgery.

[8] Deer TR, Krames ES, Hassenbusch S (2008). Management of intrathecal catheter-tip inflammatory masses: an updated 2007 consensus statement from an expert panel. Neuromodulation.

[9] Van Bommel EF, Jansen I, HendrIksz TR, Aarnoudse Aarnoudse, ALHJ ALHJ (2009). Idiopathic retroperitoneal fibrosis: prospective evaluation of incidence and clinic-radiologic presentation. Medicine (Baltimore).

[10] Koep L, Zuidema GD (1977). The clinical significance of retroperitoneal fibrosis. Surgery.

[11] Krames ES (1996). Intraspinal opioid therapy for chronic nonmalignant pain: current practice and clinical guidelines. J Pain Symptom Manage.

[12] Bidlack JM, Hemmick LM (1990). Morphine enhancement of mitogen-induced T-cell proliferation. Prog Clin Biol Res.

[13] Magazine HI, Liu Y, Bilfinger TV, Fricchione GL, Stefano GB (1996). Morphine-induced conformational changes in human monocytes, granulocytes, and endothelial cells and in invertebrate immunocytes and microglia are mediated by nitric oxide. J Immunol.

[14] Singhal PC, Shan Z, Garg P, Sharma K, Sharma P, Gibbons N (1996). Morphine modulates migration of monocytes. Nephron.

[15] Abul-Kasim K, Thurnher MM, McKeever P, Sundgren PC (2008). Intradural spinal tumors: current classification and MRI features. Neuroradiology.

[16] Vadera YS, Harrop J, Sharan A (2007). Intrathecal granuloma and Intramedullary abscess associated with an intrathecal morphine pump. Neuromodulation.

[17] Hassenbusch S, Burchiel K, Coffey RJ (2002). Management of intrathecal catheter-tip inflammatory masses: a consensus statement. Pain Med.

[18] Conti P, Pansini G, Moutachy H, Capuano C, Conti R (2004). Spinal neurinomas: retrospective analysis and long-term outcome of 179 consecutively operated cases and review of the literature. Surg Neurol.

